# Serum Adiponectin and Nitric Oxide Levels in Type II Diabetes and Its Correlation With Lipid Profile

**DOI:** 10.7759/cureus.24613

**Published:** 2022-04-30

**Authors:** Sangeeta Tuppad, Kalpana Medala, Madhusudhan Umesh, Archana Gaur, Vidya Ganji, Varatharajan Sakthivadivel, Prakash Kumar

**Affiliations:** 1 Physiology, Koppal Institute of Medical Sciences, Koppal, IND; 2 Physiology, All India Institute of Medical Sciences, Bibinagar, Hyderabad, IND; 3 Internal Medicine, All India Institute of Medical Sciences, Bibinagar, Hyderabad, IND

**Keywords:** no, endothelial dysfunction, antidiabetic, biomarkers, diabetes mellitus, adiponectin

## Abstract

Introduction

Various markers for diabetes have been identified in this new era of medicine, the most recent being adiponectin, which is primarily secreted from adipose tissue and has anti-diabetic, anti-inflammatory, and anti-atherogenic properties. It is also known to increase insulin sensitivity. Adiponectin deficiency or decreased secretion causes a variety of complications, including insulin resistance and the onset of type 2 diabetes mellitus (T2DM). One such complication of T2DM is endothelial dysfunction, which leads to decreased synthesis of nitric oxide (NO), another potent marker that normally disrupts key events in the progression of atherosclerosis.

Aims and objectives

The aim of the study was to compare and correlate serum adiponectin and nitric oxide levels with glycemic status in patients with T2DM and healthy controls.

Materials and methods

This comparative cross-sectional study included known cases of type II diabetes under group I and healthy age-matched controls under group II. Serum levels of adiponectin and nitric oxide were assessed in both the groups along with glycemic status [fasting blood sugar (FBS) and glycated hemoglobin (HbA1c)] and these parameters were compared between both groups using a t-test. Adiponectin and NO levels were correlated using Pearson’s correlation with glycemic status in group I.

Results

The mean adiponectin levels in group I were 5.94 ± 1.490 μg/mL, which was significantly (p<0.00) less than in group II, 10.30 ±1.669 μg/mL. The mean NO levels in group I (42.98 ± 6.300 μmol/L) were also significantly (p<0.00) less than in group II (56.126 ± 7.579 μmol/L). FBS and HbA1C levels were significantly higher in group I than in group II.

Conclusion

Adiponectin and NO levels were significantly reduced in individuals with T2DM when compared to healthy controls. Therapeutic interventions that increase adiponectin and NO levels may be useful targets for improving diabetes control and reducing complications.

## Introduction

Adiponectin is the cytokine secreted by adipose tissue [1]. Other tissues like osteoblasts, myocytes, epithelial cells, liver parenchyma cells, and placental tissue do secrete at a very low concentration [2,3]. Two structurally related transmembrane receptors, AdipoR1 and AdipoR2, which function as adiponectin receptors, have been identified [4]. Adiponectin has anti-diabetic, anti-inflammatory, and anti-atherogenic properties and promotes insulin sensitivity [5]. Interestingly, despite the fact that mature adipocytes secrete adiponectin, its plasma level has an inverse relationship with body fat mass. People with obesity will have lower levels of adiponectin in their blood than people of normal weight [6]. The same negative correlation is seen in type 2 diabetes mellitus (T2DM) also [7]. Adiponectin and plasma lipid levels have been linked in several studies [8]. According to the majority of research, this adipokine has an inverse association with low-density lipoprotein (LDL), triglycerides (TG), and serum cholesterol, and a positive link with high-density lipoprotein (HDL) [9,10]. Studies have shown that NO, similar to adiponectin, exhibits an inverse relationship with LDL, TG, and cholesterol and a positive relationship with HDL, which leads to atherosclerosis [11,12]. Lipid abnormalities in T2DM, also known as "diabetic dyslipidemia," are typically characterized by high LDL, TG, cholesterol, and low HDL [13].

Diabetes is one of the most catastrophic health crises of the twenty-first century, ranking among the top ten causes of death alongside cardiovascular disease, respiratory disease, and cancer [14]. According to the World Health Organization (WHO) [15], non-communicable diseases accounted for 74% of global deaths in 2019, with diabetes being responsible for 1.6 million deaths [15]. The metabolic milieu of T2DM, which includes insulin resistance and hyperglycemia, leads to endothelial dysfunction. Endothelial dysfunction has been found to be caused by changes in nitric oxide (NO) bioavailability, a rising predisposition to atherosclerosis, hypercholesterolemia, thrombosis, hypertension, stroke, and diabetes. NO is a lipophilic molecule that is principally produced in endothelial cells by three isoforms of nitric oxide synthase (NOS): neuronal (nNOS), inducible (iNOS), and endothelial (eNOS) [16]. Low concentrations of NO are favorable for various physiological and cellular functions, such as coagulation, inflammation, and maintaining vascular tone [17]. Insulin causes vasodilation and increases blood perfusion by increasing NO production [18]. Levels of NO are significantly reduced in diabetes due to oxidative stress, which leads to the impairment of eNOS [19].

Hence, the aim of the study was to compare and correlate serum adiponectin and NO oxide levels with glycemic status in patients with T2DM and healthy controls.

## Materials and methods

This comparative cross-sectional study was conducted in the tertiary care teaching hospital after obtaining approval from the Institute Ethics Committee of the Koppal Institute of Medical Sciences, Koppal, with approval number KIMS-Koppal/IEC/52/2020-21. The study was conducted between April 2020 and January 2022.

Assuming the expected population standard deviation to be 2.6 and employing a t-distribution and confidence level of 95%, the sample size was calculated as 30 using EpiTool software. All known patients with type 2 diabetes coming to the outpatient department (OPD) within the age group of 30-60 years without any other comorbidities were considered group I.

For group II, teaching faculty and nursing faculty were screened regarding their history of diabetes mellitus and any other comorbidities like hypertension, autoimmune disease, cardiovascular diseases, endocrine disorders, and obesity using a Google form questionnaire and past medical records, and those found negative for them were enrolled in the study.

Fifty patients with T2DM and fifty patients without T2DM within the age group of 30-60 years were included in the study after obtaining informed written consent. General details along with the duration of diabetes and medication history were collected through the proforma.

Basic details were collected in the proforma, which included name, age, sex, occupation, address, contact details, history of T2DM, its duration, treatment, and associated co-morbidities. Anthropometric measurements like height and weight were recorded by a stadiometer and a digital weighing scale, respectively. Body mass index (BMI) was calculated using the standard formula weight (kg)/ height (m)^2^.

After fasting for at least eight hours, venous blood samples (6-8 ml) were collected from all subjects in a vacutainer. Samples were collected early in the morning, between 6 and 7 a.m. The following parameters were estimated in both groups I and II: (i) fasting blood sugar (FBS), (ii) HbA1c (glycated hemoglobin), (iii) fasting lipid profile (total cholesterol, triglycerides, HDL, LDL, and VLDL), (iv) serum adiponectin, and (v) serum nitric oxide.

Glycaemic status and lipid profile

Fasting blood sugar was estimated by the glucose oxidase peroxidase method. HbA1c was assessed by an HPLC (high-performance liquid chromatography) based automated analyzer (Arkray, Inc., Kyoto, Japan). The lipid profile, which includes TC, TG, HDL, LDL, and VLDL, was assessed by CLIA (Chemiluminescence Immuno Assay, Siemens, Grand Prairie, TX, USA).

Adiponectin estimation 

The serum samples were separated by centrifuging the coagulated blood samples at 4000 rpm for five minutes at 4 °C, then aliquoted and stored at −80 °C until the day of the adiponectin level measurement. The serum adiponectin concentration was measured using a commercially available human enzyme-linked immunoassay (ELISA) kit.

Nitric oxide assay

Serum NO was determined indirectly by the measurement of a stable decomposition product ($\mathrm{NO_{2}^{-}}$) employing the Griess reaction. Serum samples were processed immediately with freshly prepared equal volumes of Griess reagent and incubated for 10 minutes at 37 °C. The absorbance of each sample was determined at 540 nm using a spectrophotometer.

Statistical analysis

The data were entered into Microsoft Excel 2019 (Microsoft® Corp., Redmond, WA) and analyzed using the Statistical Package for the Social Sciences (SPSS) version 22 (IBM SPSS, Armonk, NY). The mean and standard deviation (SD) were used to summarise the outcome variables. An unpaired student t-test was used to compare serum adiponectin and NO levels in groups I and II. A Pearson correlation was performed to determine the relationship between adiponectin, NO levels, and glycemic status (FBS and HbA1c). A p-value of 0.05 was deemed statistically significant.

## Results

The mean age of group I was 42 ± 5 years, and group II was 41 ± 7, which was comparable. The majority of 22 (44%) of group I were in the age group of 51-60 years. Group I included 36 (72%) men and 14 (28%) women. Among group II, the male and female participants were almost equal in number. The mean BMI of group I was 24.34 ± 2.56 with most of the patients in the overweight and obese category. In group II, most of the participants were either normal or overweight (Table 1). The FBS and HbA1c were significantly higher in group I compared to group II. Similarly, triglycerides and VLDL were found to be significantly greater in group I (p = 0.001 and p = 0.007, respectively). The adiponectin and NO values were significantly lower in group I compared to group II (Table 2). Both adiponectin and NO are negatively correlated with FBS; r = −0.848 and r = −0.769 (Table 3). Similarly, HbA1c is also negatively correlated with adiponectin (r = −0.818) and NO (r = −0.732) (Table 4).

**Table 1:** Demographic profile of participants BMI: body mass index

Parameter	Group I frequency (percentage) N=50	Group II frequency (percentage) N=50
Mean age± SD (years)	42.08±5.07	41.07±7.09
≤40	9(18)	12(24)
41−50	19(38)	16(32)
51−60	22(44)	22(44)
Gender		
Male	36(72)	28(56)
Female	14(28)	22(44)
Smokers	16(32)	8(16)
Family history of diabetes	28(56)	0
Duration of diabetes mellitus (years)		
<5	5(10)	-
6−10	7(14)	-
11−15	20(40)	-
16−20	18(36)	-
Body mass index (kg/m^2^)		
18.5−22.9	13(26)	28(56)
23−24.99	18(36)	19(38)
≥25	19(38)	3(6)
Mean BMI±SD	24.34±2.56	23.45±3.01

**Table 2:** The biochemical parameters of the patients The data are represented as mean ± SD. P<0.05 was considered significant. FBS: fasting blood sugar, HbA1c: glycated hemoglobin, TC: total cholesterol, TRI: triglycerides, HDL: high density lipoprotein, LDL: low density lipoprotein, VLDL: very low-density lipoprotein, NO: nitric oxide.

Parameter	Group I, n=50	Group II, n=50	p-value
Age (years)	42.08 ± 5.07	41.98 ± 4.57	0.918
FBS (mg/dl)	191.30 ± 70.53	89.88 ± 6.49	0.000*
HbA1c	7.94 ± 0.74	6.20 ± 0.57	0.000*
TC (mg/dl)	178.70 ± 31.85	167.48 ± 29.53	0.071
TG (mg/dl)	238.74 ± 83.95	191.72 ± 55.02	0.001*
HDL (mg/dl)	43.64 ± 9.10	45.04 ± 6.05	0.367
LDL (mg/dl)	88.50 ± 33.03	83.28 ± 28.18	0.397
VLDL (mg/dl)	45.38 ± 16.82	37.84 ± 9.60	0.007*
Adiponectin (μg/mL)	5.94 ± 1.49	10.30 ± 1.66	0.000*
NO (μmol/L)	42.98 ± 6.30	56.126 ± 7.57	0.000*

**Table 3:** Correlation of adiponectin with demographic data and glycemic status

Parameters	r	p-value
Age	0.187	0.193
Sex	−0.033	0.821
BMI	−0.119	0.412
FBS	−0.848^*^	0.000
HbA1c	−0.818^*^	0.000

**Table 4:** Correlation of NO with demographic data and glycemic status

Parameters	r	p-value
Age	0.044	0.761
Sex	−0.054	0.709
BMI	0.053	0.276
FBS	−0.769^*^	0.000
HbA1c	−0.732^*^	0.000

## Discussion

In the current study, we assessed adiponectin and NO levels in patients with type 2 diabetes mellitus (group I) and people without type 2 diabetes mellitus (group II) and found that adiponectin levels and NO levels were significantly lower in group I when compared to group II, and there was a significant negative correlation between adiponectin, NO levels, and FBS in group I.

We found in our study that age had no statistically significant correlation with adiponectin because the study included only a narrow age range. As evident from various studies, adiponectin levels increase with age, and this may be explained by the fact that the decline in sex steroidal hormones with age tends to raise adiponectin levels in the elderly [20,21]. In addition, we discovered that females had higher levels of adiponectin than males, but this was not statistically significant. The narrow age range in the current study may be the reason for this. The following factors could account for the gender difference in serum levels of adiponectin: (i) androgens have an inhibitory effect on adiponectin secretion [22], (ii) the difference in the distribution of fat cells in males and females: adiponectin production may be influenced by the number and size of fat cells [23]. Unlike other adipose-derived hormones like leptin and resistin, which correlate positively with obesity measures, adiponectin correlates inversely with obesity in rodents and humans [24]. Studies have shown that anthropometric determinants of obesity like waist circumference (WC), hip circumference (HC), and BMI (body mass index) are negatively correlated with levels of adiponectin. In the current study, BMI was negatively correlated with adiponectin but was not statistically significant [25,26]. Adiponectin regulates insulin sensitivity, increases fat metabolism, regulates glucose tolerance, and modifies homeostasis in order to protect people from diabetes, which makes it the strongest biomarker for type 2 diabetes mellitus [27]. In the current study, adiponectin levels were significantly decreased in patients with type 2 diabetes mellitus when compared to healthy controls, which was in line with various other studies [28,29].

Even though our study did not have a significant correlation between age and NO levels, in healthy human beings, enzymatic NO production reduces progressively with age, according to studies. The use of techniques to identify and treat NO inadequacy may be extremely beneficial to the geriatric population [30].

In the current study, the NO levels in patients with type 2 diabetes were significantly reduced when compared to the controls, which was similar to the majority of the studies [31,32]. The possible reason is endothelial dysfunction, which is a common sequel of diabetes, leading to impaired endothelial nitric oxide synthase (eNOS) activity, causing decreased NO production [32]. Hyperglycemia impairs endothelial function through increased oxidative stress which is a hallmark of diabetics [33]. Hyperglycemia accelerates the formation of advanced glycosylated end products, boosts the polyol pathway, and activates protein kinase C, resulting in oxidative stress [34]. Diabetic individuals have a lower glutathione concentration, which is an essential antioxidant in erythrocytes. Furthermore, decreased radical-trapping antioxidant parameter (TRAP) levels and increased lipid peroxidation levels suggest the presence of elevated oxidative stress in diabetes in vivo [35]. Reduced NO availability may be relevant not only to the development of atherosclerotic problems in T2DM but may also interfere with insulin-mediated postprandial glucose clearance and contribute to the development of insulin resistance [36]. Also, NO disrupts key events in the progression of atherosclerosis, including vascular tone, monocyte and leukocyte adhesion to the endothelium, platelet-vessel wall interaction, and smooth muscle proliferation. As a result, the pathogenesis of diabetic vascular disease may involve a decrease in endothelial-derived NO bioavailability [37].

Triglyceride and VLDL levels were also found to be significantly higher in group I, while HDL was lower. Lipid abnormalities in diabetes are called diabetic dyslipidemia, which is associated with excess hepatic production of VLDL. Increased amounts of fatty acids returning to the liver in diabetic patients are reassembled into triglycerides and secreted in VLDL. Increased plasma VLDL concentrations cause the exchange of triglycerides from VLDL for cholesterol esters found in HDL, which can account for low HDL levels [38]. Studies in people with type 2 diabetes have found an increased association between CAD and high triglycerides and low HDL levels [39-41].

Limitations

Because the sample size in our study was limited, the exact association could not be determined; thus, larger-scale investigations with a higher sample size might shed additional light on the relationship between NO, adiponectin, and T2DM.

## Conclusions

Our work was a basic attempt to study the association between adiponectin, NO, and T2DM. Adiponectin levels in T2DM were significantly lower. Although various studies have shown similar results, they have failed to establish the consistency of the association across diverse populations. Future research should look at whether adiponectin, in addition to existing risk variables, might help predict T2DM using prognostic statistical approaches. Our data suggest that NO concentrations are significantly lower in type II diabetes, lending credence to the concept that an altered NO pathway is a key defect that leads to T2DM. However, further research is needed to acquire a better knowledge of the critical events involved in creating and demonstrating the significance of adiponectin and NO assays in T2DM.
